# “Someone catching me when I’m falling”: a qualitative study exploring satisfaction in romantic relationships where one partner has borderline personality disorder

**DOI:** 10.3389/fpsyt.2026.1807149

**Published:** 2026-07-20

**Authors:** Emily Molyneux, Jillian Helen Broadbear, Sathya Rao, Karthika Kasiviswanathan, Sadaf Zafir, Tess Knight, Helen Mildred

**Affiliations:** 1 School of Psychology, Deakin University Melbourne Burwood Campus, Melbourne, VIC, Australia; 2 Spectrum Personality Disorder and Complex Trauma Service, Research and Innovation, Richmond, VIC, Australia; 3 Eastern Health Clinical School, Monash University, Melbourne, VIC, Australia; 4 School of Clinical Sciences, Monash University, Melbourne, VIC, Australia; 5 Medicine, Nursing and Health Sciences, Monash University, Melbourne, VIC, Australia; 6 School of Psychology, The Cairnmillar Institute, Melbourne, VIC, Australia; 7 Infant, Child, & Youth Mental Health Service, Mental Health & Well Being Program Research & Evaluation Unit, Eastern Health, Melbourne, VIC, Australia

**Keywords:** secure attachment, borderline personality disorder, open communication, romantic relationship, relationship satisfaction, lived experience, qualitative research, emotion regulation

## Abstract

**Introduction:**

Interpersonal difficulties are a core challenge experienced by people who have borderline personality disorder (BPD). Relationships where one person has BPD are often rated lower in satisfaction than others due to their tumultuous and unstable nature. Interpersonal difficulties not only cause distress but may also catalyse crises for people with BPD. The ability to sustain emotionally positive attachments is fundamental for recovery in BPD. The aim of this study was to understand how couples, where one partner has BPD, experience their intimate relationships and make sense of their success.

**Method:**

Through semi-structured interviews, we explored the role of attachment in the context of these romantic relationships and identified constructive behaviours that contribute to their longevity and satisfaction. In-depth interviews were conducted individually with six couples engaged in long-term (>1 year) relationships.

**Results:**

Interpretative Phenomenological Analysis (IPA) was used, and two key themes were identified – a) navigating the impacts of the disorder and b) safe base and secure attachment.

**Conclusion:**

Our findings suggest that reorientation of interpersonal transactions, where the partner without BPD serves as a stable base, enables open communication. As a result, people with BPD feel secure, promoting emotion regulation, remission from symptoms, and an ability to sustain a long-term, satisfying relationship.

## 1 Introduction

Borderline personality disorder (BPD) is a complex mental health condition characterised by intensity in interpersonal relationships, emotional dysregulation, and unstable mood and self-concept (1). Around 1.8% of the community experiences BPD (2). Despite the lack of significant sex-based differences in community prevalence, 75% of people diagnosed with BPD in clinical settings are women (3, 4).

BPD is commonly described as developing from an interaction between a child’s biological vulnerability to experiencing heightened emotions and repeated exposure to an invalidating environment (5). This leads to considerable distress and suffering and contributes to interpersonal instability. Early-life adversity is commonly noted in people diagnosed with BPD. For some, this includes experiencing dangerous, abusive, or neglectful relationships in childhood from adults, often including caregivers (6). This view aligns with the attachment theory proposed by John Bowlby – a psychological and evolutionary framework – which posits that infants have an innate biological drive to form a strong attachment to their caregivers to ensure survival (7). It emphasises the role of secure parent–child attachment in fostering confidence to rely on the primary caregiver for comfort and support, including the development of emotion regulation and interpersonal capacities (7). From this perspective, BPD symptomatology is often conceptualised as attachment-related, whereby problematic early caregiver relationships may contribute to the development of insecure attachment styles (anxious/avoidant) (8–10). In the context of insecure attachment styles, people with BPD often report patterns of maladaptive coping behaviours as responses to heightened relational distress (11–14).

Attachment styles tend to remain relatively stable across the lifespan and influence patterns of interaction within romantic relationships (15). Avoidant attachment is typically associated with self-reliance, fear of intimacy, and distrust of others; anxious attachment is associated with fears of abandonment and heightened needs for intimacy and reassurance (16, 17). In contrast, secure attachment is linked to open communication, emotional safety and confidence in a relational context (12, 15). Within securely attached relationships, individuals are more likely to elicit secure base behaviours such as clearly communicating needs, responding sensitively to a partner’s distress, and providing appropriate support (18).

Attachment processes underpin many functional aspects of romantic relationships during their formation, maintenance and dissolution (15). Interpersonal dysfunction remains one of the most pervasive and impairing features of BPD, often manifesting in intense relational instability, oscillations between idealisation and devaluation, frantic efforts to avoid abandonment, and recurrent conflicts (1, 19–23). Consistent with this, many people with BPD report histories of tumultuous romantic relationships characterised by high intensity of positive emotions that contrast with hostility, demand-withdraw communication patterns and maladaptive conflict resolution strategies such as dominance, blame, and criticism (19–23). These relational difficulties are a significant source of emotional distress for people with BPD, with ruptures prompting suicidality, self-harming behaviours and negative consequences for intimate partners (11, 20, 22–24).

By contrast, stable intimate relationships are a powerful protective factor associated with remission and recovery. Despite the centrality of interpersonal difficulties in BPD, some people with BPD report having satisfying, long-term relationships (25). Evidence suggests that approximately 79% of people with BPD who have recovered are married or live with a romantic partner compared with 39% of people with BPD who are yet to recover (26), strongly suggesting a correlation between healthy romantic relationships and symptomatic remission. When people with BPD are asked what recovery means to them, their perceptions extend beyond symptom reduction, highlighting improvement in the quality of interpersonal connections (27, 28).

The beneficial nature of healthy romantic relationships and their importance to people’s well-being underscore the importance of examining how attachment style can affect interpersonal aspects of BPD. Attachment theory is a social model of relational beliefs that reflects upon how one comes to view oneself and others (7). Differences in attachment styles (either secure or insecure) may feature in couples where one person has a diagnosis of BPD. Such differences could manifest in relational areas such as conflict and mutual caregiving, negatively impacting relationship quality and satisfaction (15, 21, 29). However, satisfaction in romantic relationships can be influenced by the partner without BPD, referred to as ‘partner effects’ (15, 30). For example, engagement in ‘secure base’ behaviours by the person without BPD can assist the partner with BPD to regain emotional stability (18). Conversely, behaviours characteristic of insecure attachment by the partner without BPD—such as withdrawal of intimacy, misattunement or invalidating responses to distress, demand–withdraw communication patterns, or reactive responding—may exacerbate dysregulation and contribute to high levels of distress in the partner with BPD (31, 32). Although previous literature has established links between attachment processes and BPD (10), or links between attachment and romantic relationship functioning (22, 25), comparatively few studies have examined attachment dimensions specifically in the romantic relationships of people with BPD.

Extant qualitative studies have explored the subjective experiences of people with BPD navigating healthcare, treatment, and recovery (27, 33–35). Perspectives of parents living with a child with BPD have also been reported (36, 37). However, there is limited research investigating characteristics and protective factors associated with healthy romantic relationships in people with BPD. While one study reported on relationship conflict in people with BPD (38), constructive behaviours employed during conflict were not studied.

To our knowledge, this is the first study that brings a strength-based approach to exploring factors contributing to the success of a romantic relationship in couples where one partner has BPD. The aim of this study was to understand ways in which romantic relationships, where one partner has BPD, are satisfying for couples who have been together for at least one year. The research questions were: a) How do couples, where one partner has BPD, fulfil attachment functions for one another, such as providing a safe space when distressed or in conflict, and a secure base from which to grow and develop psychologically? b) What are the strengths and positive features that contribute to relationship duration and satisfaction?

## 2 Materials and methods

Interview data were collected and analysed using interpretative phenomenological analysis (IPA), which allowed each participant to make sense of their lived experience (idiographic) and researchers to develop a broader understanding of the topic (hermeneutic) through interpretation of participants’ experiences (39). The authors adhered to the standards for reporting qualitative research (SRQR) for each section of this paper (40). Gendered language throughout is based on each participant’s reported gender.

### 2.1 Sampling and data collection

Couples were recruited through advertisements on the websites and social media pages of a national consumer and carer organisation, and a specialist service for personality disorders. Flyers were also posted in the waiting room at the study site.

Inclusion criteria were participants aged 18+ years who were fluent in English and engaged in a romantic relationship lasting at least one year at the time of the study, where they or their partner had a formal BPD diagnosis. Both partners were required to have rated their relationship as at least ‘moderately satisfying’, using the Perceived Relationship Quality Components (PRQC) screening tool (41). Research suggests that couples in romantic relationships relate to one another as a ‘safe haven’ after four months of being together but take two years to perceive them as a ‘secure base’ (42). Hence, a conservative duration of one year was chosen as the minimum to satisfy the definition of a ‘long-term relationship’ for our study.

Seventeen people who either had BPD or had a partner with BPD completed an initial online screening survey (Qualtrics© 2020, Provo, UT) before completing the PRQC (internal reliability of α = .88) (41). A plain language participant information sheet accompanied the screening survey. Of the 17, five people did not meet the study inclusion criteria, and six couples (n=12) consented, were contactable, met the inclusion criteria, and could attend an interview in person or by telephone. All six couples were provided with a copy of the questions before their interview. The first author met with each couple together to explain the study, respond to any questions, and confirm consent from all twelve participants.

Interviews with each member of the couple were then conducted one after the other. Internal confidentiality was addressed by ensuring that all six couples were aware of the focus of the interview and the study process, including the questions that would be asked and the option to skip questions (43). They were also informed that the results of the study would be published at a later stage. All six couples were heterosexual; participants with BPD were all female. Underdiagnosis of BPD amongst men possibly played a role in the makeup of our sample (44). Five of the six couples had been together for 2–10 years, with one relationship of 18 months. The mean participant age was 31.75 (± 6.55). Further demographic information is included in the Supplementary Materials.

### 2.2 Procedure

The project received institutional and university ethics approval. Interviews were in-person except for one couple who lived interstate. They were interviewed separately over the telephone. All participants received a $25 gift card in appreciation of their time.

The interview guide (Supplementary Materials) was developed by the research team based on the principles of IPA, with open-ended questions that covered relevant topics and encouraged understanding of each individual’s experience contextually, and the meaning they attached to these experiences (45). Where appropriate, participant responses were explored using additional questions (45).

All interviews, except one, lasted 40–50 minutes. The shortest interview (20 minutes) occurred for one participant without BPD but with an ASD diagnosis, who spoke very concisely. Interviews were electronically recorded and transcribed by hand. Each participant had the opportunity to review their transcript. All interviews were conducted by the first author, who is in a stable and long-term relationship, works clinically with people who have a diagnosis of BPD, and whose interest in this research stems from her curiosity to hear the perspectives of partners of people with BPD. The reflexivity of other authors is described in the Supplementary Materials. Pseudonyms are used throughout to maintain confidentiality.

Following the IPA protocol (45), each recorded interview was listened to by the first author while considering the participants’ intended meaning(s). Each transcript was read and re-read by the same author, with initial exploratory notetaking about content and language. This was followed by identifying conceptual comments and developing possible codes and themes across each case. To ensure rigour, four transcripts (33.3%) were independently analysed by the rest of the analytic team. The authors met several times to discuss patterns (codes and themes) across cases and form a broader understanding of participants’ experiences. Consensus was reached on the overarching themes and subthemes. Consistent with IPA, themes were compared across cases for convergence and divergence, with themes present in at least half of the sample being selected. Due to time and resource limitations, member checking was not done. However, while analysing the transcripts, the authors maintained a reflexive approach through regular debriefings during discussions.

### 2.3 Measures

Participants also completed the Attachment Style Questionnaire – Short Form (17), a comprehensive assessment of adult attachment. This revealed that none of the study participants, regardless of having BPD, had a secure attachment style. Details related to this measure are presented in Supplementary Materials.

## 3 Results

The IPA data analysis generated two key themes and *five* subthemes (**Table 1**). The key themes were – a) navigating the impacts of the disorder and b) safe base and secure attachment. Representative quotes that guided the development of themes and sub-themes are included in the following sections under each theme. **Table 2** clarifies which pairs of participants are a couple.

**Table 1:** Key themes and sub-themes.

1. Navigating the impacts of the disorder 1.1. Emotional and relational challenges 1.2. Utilisation of emotional regulation and interpersonal skills 1.3. Communication to bring understanding
2. Safe base and secure attachment 2.1. Recognition of mutual commitment and care 2.2. Partner serving as a safe base

**Table 2:** Participant dyads.

Dyad no.	Partner with BPD (BPD)	Partner without BPD (w/o BPD)
1	Josie	Dylan
2	Tina	Sean
3	Donna	Paul
4	Lisa	James^1^
5	Sarah	David^2^
6	Casey	Brent

1 Diagnosis of ASD.

2 Diagnosis of ADHD.

### 3.1 Navigating the impacts of the disorder

Partners of participants diagnosed with BPD spoke about some of the most challenging features of the disorder and how it affected their lives, relationships, and their ability to provide support. One of the key challenges they discussed arose when their partner became emotionally dysregulated. Participants also discussed how they navigated these challenges by utilising emotional regulation and interpersonal skills, which improved their relationship quality.

#### 3.1.1 Emotional and relational challenges

Male partners without BPD described witnessing their partner’s emotional dysregulation in response to situations that they themselves perceived as trivial. They shared how this led to tension and unpredictability in their relationships. One male partner described their partner as having a “disproportionate” emotional response to “something small.” Similar feelings were expressed by another, stating that at times his partner’s “response wasn’t equal to what was going on”, highlighting the impact of sensitivity and reactivity associated with BPD on romantic relationships. Another reported finding these outbursts “jarring,” with their suddenness sometimes leaving him reeling: “… sometimes the smallest things I do creates a large reaction, which I’m never ever going to be ready for (laughs)! Quite often things that I consider very small, she does not, and that is very frustrating for me…”

Although the male partners without BPD perceived these reactions as “disproportionate,” all study participants rated their relationship as moderately satisfying. One explanation is that partners without BPD were able to navigate the emotional sensitivity of partners with BPD who struggled to regulate their emotions in moments of distress. For example, one male participant stated that he was able to be curious about his partner’s reactions rather than feel distressed: “I try to think of why this is occurring … and understand why that might be … it’s not the thing that she’s upset about, but there’s something behind that driving it, so [I] try and get some kind of insight.”

This partner’s self-described “analytical” nature seemed to assist him in creating some space to process his own response to her behaviour, enabling him to take it less personally and interpret the reactions of his partner with BPD differently. This male partner without BPD shared:

“…when you’re in a relationship with someone with BPD, that conflict exists on a scale that is much higher than usual, but it’s still coming from the same place … it’s been helpful to try and understand that it’s a super emotional response to a problem that’s actually in some cases legitimate … or reasonable.”

This reflects the importance of ‘partner effects’ in the sustenance of satisfying romantic relationships where one partner has BPD. His use of the words “super emotional response” and “legitimate” exhibits his ability to mentalise his partner’s context, which may arise from her insecure attachment style.

In contrast, James (D4; w/o BPD), with a diagnosis of autism spectrum disorder (ASD), and David (D5; w/o BPD), with a diagnosis of ADHD, appeared to struggle with their partners’ strong emotions. James described trying to understand Lisa (D4; BPD) and figure out “if she’s having a bad day,” and David described wanting to act to remedy his partner’s “turns of emotion.” On the other hand, Sarah (David’s partner, D5; BPD) expressed awareness of her partner’s difficulty and reported having learned to communicate her needs very clearly.

Participants without BPD shared their experience of coping with the symptoms of the disorder in their partner and the heavy emotional toll it sometimes took on them both. They shared how their partners’ self-injurious behaviours impacted their own mental health, causing some to question their ability to cope and remain in the relationship.

Sean (D2; w/o BPD) reflected that “in those first couple of years, things just got worse and worse and worse” when “[Tina] was bouncing around the [mental health] system,” which caused him to become overwhelmed. His partner, Tina (D2; BPD), reflected that – “it didn’t seem like I was ever going to change or improve … and the fear that he may not be able to be steadfast … really broke him apart and he became sort of unwell.” The phrase “the fear that he may not be able to be steadfast” reflects Tina’s understanding of Sean’s commitment. The couple recognised this breaking point in their relationship and worked towards remedying it. Sean went away for a few weeks, which helped him regulate himself, and Tina learned to “reflect before reacting”. Sean described – “she’s a lot better at being able to self-regulate … like if she’s getting agitated, she’s able to pick that up a bit sooner.” This indicates the role of intimate relationships in motivating people with BPD to engage in their own recovery.

Another male partner, Brent (D6; w/o BPD), spoke about self-care as he admitted that earlier in the year, he felt he had “reached [his] limit” and that:

“…it’s hard, it’s a bit like PTSD in a way, like that kind of trauma can persist after the events in me, not just in her, so I think the biggest challenge going forward is my self-care so that I’m available to support her.”

He also described carrying the weight of responsibility, saying: “it can be troubling for me … because I don’t really have the resources to help me out. I would like somebody to talk to.” Despite numerous strengths to their relationship, he admitted that sometimes he sought respite in his own home away from his partner during crises. The “biggest challenge” expressed by partners without BPD was self-care and identifying “resources” for coping so they could be “available” for their dysregulated partner. They reported experiencing vicarious trauma and the importance of identifying external supports for carers and partners of people with BPD.

Despite difficult times and challenging experiences, all male participants remained deeply committed to their partners. Five of them listed the strengths in their relationships and judged them to outweigh the hardships. The mutual commitment (“something to work for”) observed in these relationships appears to contribute to the satisfaction and longevity reported by the participating couples; however, access to support for partners without BPD would lead to further improvements.

#### 3.1.2 Utilisation of emotional regulation and interpersonal skills

Four partners without BPD perceived gradual improvements in their partners’ ability to regulate their emotions and practice interpersonal skills. They reported fewer arguments, breakups, or threats of breakups. Narratives in this subtheme reflect changes observed in the male partners as their partner with BPD learned to regulate their emotions.

Partners without BPD – Sean (D2), Dylan (D1), Brent (D6), and Paul (D3) – reflected on their use of a range of skills as couples, which increased their partners’ self-efficacy. Paul said, “…either she can calm down to the point where she can sleep, or I am able to help regulate her…” Sean knows he can “calmly go ‘it’s okay, it’s okay’, and try to de-escalate her as best as I can.” He acknowledged “that’s another thing that took time” for him to master because his “natural instinct [was] to yell back at her to stop yelling.” Tina (D2; BPD) validated his assertion and said, “…if I have a problem with something, he’ll just calm me down first, and then we will talk about whatever the problem is.” She explained how Sean initiated new relationship patterns for her and how his treatment of her had changed how she responded:

“…he doesn’t react the same way as or even treat me the same way as I was treated previously, and I’ve always wondered why, and analysed the differences, differences between Sean and … previous relationships, and … how I respond to the differences (laughs)”

Resilience built through trial and error (“natural instinct [was] to yell back”) in male partners provides a space for the partner with BPD to engage in change, such as learning more about their interpersonal triggers, understanding heightened emotions, and reflecting before reacting. Participants with BPD reported using “structured conversations” with their partners, recognising its utility for communicating their needs and resolving conflict. Josie (D1; BPD) explained:

“…we would even sit down with the DBT [Dialectical Behaviour Therapy] book and … we’d go through the structured conversation [section] trying to resolve the problem. …luckily, Dylan is a very good communicator, and I’ve grown into one, I guess from DBT.”

Dylan’s (D1; w/o BPD) personal quality of being a good communicator seemed to influence Josie’s communication abilities (“I’ve grown into one”).

Paul (D3; w/o BPD) and Dylan (D1; w/o BPD)’s willingness to receive feedback from their partners likely influenced their partners’ ability to openly communicate with them (“she’s able to actually say”), reiterating the importance of ‘partner effects.’ Their willingness suggests a secure sense of self and an understanding of their contribution to the relationship dynamic. As partners with BPD made progress on their recovery journey, the men felt hope, and both partners recognised their own ‘role’ in the recovery process.

Couples shared the importance of DBT in facilitating emotion regulation. Josie (D1; BPD) felt that:

“DBT itself, it just makes such a big difference … I realised just how many skills I just didn’t have in everyday life … I’d be coping with things like I was a kid, and I didn’t realise that was not normal until I did DBT…”

Josie’s (D1; BPD) use of the phrase “like I *was* a kid” indicates her recognition of her growth. Dylan (D1; w/o BPD) actively practices DBT skills with Josie and reminds her of skills to employ when she needs them, “he will sit with me … he’ll be like ‘I think you’re struggling with this right now, why don’t we try this skill from DBT….” (Josie). Dylan’s ability to identify Josie’s needs by saying, “I *think* you are struggling with this”, reflects his attunement to her. He reasons with Josie (“Why don’t *we* try this skill?”) and assists her in practising the skills by acting as her secure base. Other couples also used DBT to create a pause for ‘mindfulness’ and ‘different action’ when they sensed escalation. They found it helpful to have a short time apart, calm their emotions and come together later to reconnect and “try again” (Dylan).

Donna (D3; BPD) said she and Paul (D3; w/o BPD) often “try to get a little pause … and then have a conversation when we’re both in the place where we’re not very emotional anymore.” Sean (D2; w/o BPD) indicated that Tina (D2; BPD) “knows how to step back and breathe when her anxiety is starting to creep up.” Sarah (D5; BPD) acknowledged:

“Something I have been learning and trying to practice more and more is having the pause between the ‘getting angry’ and ‘doing the angry thing’, and there are times when I don’t do that great … I’m still working on making my pause bigger.”

Couples recognised each other’s needs (“we’re *both* in the place”) and acknowledged their mistakes (“there are times when I don’t do that great”), indicating a conscious effort (“*try* to get a little pause”) for maintaining a healthy romantic relationship.

#### 3.1.3 Communication to bring understanding

All couples highlighted the role of open communication as a hallmark that contributes to relationship longevity. Brent (D6; w/o BPD) and Casey (D6; BPD) live separately for financial reasons, and Brent explained that they’re “always in communication” with each other. Josie (D1; BPD) and Dylan (D1; w/o BPD) also felt communication was the “main strength” of their relationship. Dylan attributed low levels of conflict and relationship longevity to communication - “because we talk about everything, …it has meant we’re able to last this long.” The couples valued their partner’s differing views. Donna (D3; BPD) explained that she and Paul (D3; w/o BPD) “spend quite a lot of time … really talking about … how it feels for each other.” Similarly, Lisa (D4; BPD) said, “We try really hard to understand the other’s point of view.” Participants’ efforts to “try really hard to understand” differing views reflect their understanding of constructive communication and its importance in sustaining a relationship. Tina (D2; BPD) believed that:

“It’s normal for each of you to push your point, and when we see that one of us is becoming more heated in pushing that point, the other will question and wonder why the person is becoming so heated, then it doesn’t become necessarily about what the disagreement is, but also about why the person is becoming so upset, and we address that.”

Couples described understanding each other’s context (“why the person is becoming so upset”) which played a crucial role in conflict resolution.

Four couples described rules that they had developed for managing conflict based on reflections from past challenges. Lisa (D4; BPD) said, “We … have a rule that if we’ve had an argument, we must say sorry or come to a reconciliation so that it doesn’t continue for the next day.” Sarah (D5; BPD) and David (D5; w/o BPD) have devised similar scripts that help them help each other. Sarah explained:

“David has an ability to go ‘what you have said has hurt me’, or ‘what you’ve done has affected me in this negative way’, which is the sentence that I need to be able to go ‘okay, now I’ve overstepped’ or ‘what I’ve done is not okay’.”

These rules bring a sense of safety (“doesn’t continue for the next day”) and set boundaries surrounding what’s acceptable (“what I’ve done is not okay”). Donna (D3; BPD) stressed the importance of mutual trust by saying, “We have created some … boundaries, …and I really, really trust him to be there for me in those times, and act in a way that we have agreed beforehand.”

Creating rules seemed to help resolve conflicts and stemmed from a willingness to be open about their needs and desires. When followed, these frameworks for success created patterns of improved communication and trust over time.

Four of the six couples also described discoveries through trial and error that soothed their partners when they were stressed or upset. Paul (D3; w/o BPD) described: “…there’s no magic bullet, but if something works, yeah, then do that again.” Three couples discussed how physical touch is sometimes “more effective than words” (Donna, D3; BPD). Paul finds touch to be most useful “when there might be extreme conflict … I do that even though she’s often saying things that wouldn’t encourage you to hold someone.” Hence, by initiating and maintaining closeness with his partner at times of distress, Paul can help regulate his partner’s emotions. Similarly, Sarah (D5; BPD) said:

“I sent him [David, D5; w/o BPD] a set of pictures once that’s about how to care for someone who’s not well, and it’s called making someone into a sushi roll. You roll them in a blanket and stick them on the couch, and then you bring them hot drinks! And so, whenever I’m not well, he goes, “I can make you into a sushi blanket?” and he’ll wrap me up.”

Sarah reported this as comforting and used it to elicit care from David (D5; w/o BPD), who sometimes struggled to know how to help her. Josie (D1; BPD) explained: “I need him to be still with me, like hold me firmly or something … I had to learn how to tell him what I needed at the time … I didn’t want to have to tell him. I wanted him to automatically do it.”

Even though participants found it difficult (“I didn’t want to have to tell him”) to openly communicate and express their emotions through words or actions (“I do that even though she’s often saying things that wouldn’t encourage you to hold someone”), they were able to recognise the benefit of open communication in successful conflict resolution and emotion regulation. This enabled them to get out of their comfort zone and communicate their needs clearly to their partners, which addressed their distress through connectedness instead of hostility.

### 3.2 Safe base and secure attachment

Some male participants described ways of acting as a safe base for their partners with BPD. They revealed perspectives relating to a key feature of their relationships, assisting them in creating a secure attachment with their partner, i.e. recognition of mutual commitment and care.

#### 3.2.1 Recognition of mutual commitment and care

Five couples recognised their mutual commitment to the relationship and one another’s well-being. Josie (D1; BPD) told Dylan (D1; w/o BPD) about her diagnosis on their first date, which encouraged mutual commitment. Dylan explained, “We decided very early on to stick through everything … because of the fact that she had BPD, she needed someone who’s solid.” They identified that a strong sense of commitment would help Josie feel safe and that the relationship could provide an environment where she could practice skills to combat her interpersonal triggers.

Tina (D2; BPD) described Sean (D2; w/o BPD) as “very dedicated” to her when he became very “involved in [her] diagnosis and care.” Similarly, Sean felt Tina “stepped up” to support him when he had his “breakdown.” Sean commented, “Okay, one of us is struggling, but the other one can, you know, pick up or hold them up while going through it…” Donna (D3; BPD) described her and Paul (D3; w/o BPD) as both being “very dedicated to improving the relationship … and to learning to be a better partner…” They discussed what they needed from each other and felt equally committed to the relationship and treatment. Paul acknowledged Donna’s “commitment to do the work,” and that she expected him to “apply the same effort and commitment.” Honest discussions about each other’s struggles seemed to foster commitment. Couples openly communicated their commitment to the relationship through words or actions, contributing to ongoing dedication and care for one another.

Relatedly, five couples expressed expectations for their partner to try as hard as they themselves do, both trying to grow and learn within the relationship. Sarah (D5; BPD) explained:

“I think in past relationships I’ve had partners who’ve been … willing to just let me be unwell. … he’s come from a family where you just get up and you do it, and it took a while for him to adjust to knowing when I couldn’t do something, and when I just really didn’t want to do something … But that has helped immensely having someone go ‘no you can get up and you can do this, you need to just push through,’ at the right time, but also going ‘no that’s okay you can’t’ …we will get up and try again tomorrow.”

David’s (D5; w/o BPD) ability to be logical likely helps Sarah (D5; BPD) access the DBT skill of ‘wise mind’ and make more balanced choices when she is overwhelmed by her own ‘emotional mind.’

All couples recognised that their mutual care for one another promoted a sense of equality in the relationship. Sarah (D5; BPD) looked after David (D5; w/o BPD) when he had surgery on his knee. Shortly after that, she had surgery, and “…he helped look after [her]. It was very nice, taking care of each other (laughs).” The couples also described doing kind acts for one another simply to “take the stress off” each other (Sarah). Sarah shared that she took on house responsibilities when David was stressed which “really help[ed] him a lot.”

Josie (D1; BPD) explained that they try to “both put the opposite person first.” She recalled Dylan (D1; w/o BPD) had cleaned the house while she was unwell: “he knows that just little things like that make a big difference to my mental health.” These acts of service seem to communicate commitment and care which can foster trust and relationship security, contributing to relational maintenance.

#### 3.2.2 Partner serving as a safe base

Nine participants used language that indicated they believed their partner to be trustworthy, attuned, and capable of helping when they felt distressed. Donna (D3; BPD) felt that Paul (D3; w/o BPD) “can deescalate it just by being there for me … and just putting his own emotions aside….” She described him as “non-judgmental” and “trustworthy”, and as “…sort of, someone catching me when I’m falling.” These words exemplify Donna’s secure attachment to Paul and her confidence in him to provide comfort and security when she is overwhelmed. Tina (D2; BPD) described Sean (D2; w/o BPD) as someone with “an unwavering resilience, and very deep patience, and understanding and compassion,” and is “very solid.” She said that they went through an “awful incident” that was “…really quite cataclysmic, and it would have been enough to turn a lot of people away, and Sean just didn’t.” Like Paul (D3; w/o BPD), Sean (D2; w/o BPD) was able to remain focused on supporting Tina (D2; BPD) despite his feelings of anger or frustration.

Dylan (D1; w/o BPD), Paul (D3; w/o BPD), and Sean (D2; w/o BPD) all spoke about how they see their partner as a person before they see the diagnosis and acknowledged that while important, this can be challenging at times. Dylan shared:

“She [Josie, D1; BPD] kept asking me when we first started dating “Why are you with me?” And I’d say because …. I can see through the fact that she has BPD and see that she’s actually a really nice person underneath all that. … I’ve been told that I come across not very well on first encounters, but I’m not actually like that (laughs) underneath.”

Dylan’s (D1; w/o BPD) ability to reflect on his own experiences of being misjudged enabled him to empathise with Josie (D1; BPD). He remarked: “The thing that helped her a lot was the fact that I wouldn’t really react (laughs). I was like, you’re not you when you’re like this … and I’m just going to wait until it’s over. “Dylan “not reacting” and “separating it” made an enormous difference to Josie:

“He’s always made a point that he sees me as separate to the diagnosis, and that’s always been really beneficial to me because … no one had ever broken it down like that for me … it’s always been like “why are you being so difficult” … “why are you so angry, why do you have to be like this” …and Dylan … just separated the two.”

Notably, according to the ASQ-SF, Dylan’s attachment anxiety was higher than Josie’s, and his desire to stay with her may reflect his preference to move towards her rather than away. In contrast to this, David (D5; w/o BPD) scored higher on avoidant attachment than Sarah (D5; BPD) did. When asked about how he contributes to their relationship success, he remarked, “I hadn’t thought about that. Huh, that I personally contribute.” He did not speak about being involved in aspects of Sarah’s treatment but recognised that doing so would improve their relationship positively.

Paul (D3; w/o BPD) acknowledged that while he tries to hold to the view that Donna (D3; BPD) is separate from her BPD, when her words are harsh, “it’s very hard to separate the disorder from the person because it is the person who is saying that to you…” He perceived that she became someone different when she was dysregulated and, in those times, he reminded himself of the person he loved. He explained how the concept of a social model helped him to increase his support for Donna and take ownership within his relationship: “…the transactional model was kind of a light bulb moment... Whatever the challenges that exist in the relationship with the person with the disorder, 50% of them are …the support person. … they exist in that transaction.”. Paul learned and practised DBT skills with Donna because he believed it could “change that transactional model with the person who is suffering.” He stated that Donna “often explains very clearly how I [support person] can change my behaviour and make it better for her…”

Similarly, Dylan (D1; w/o BPD) acknowledged the importance of him being “stable” for Josie (D1; BPD) and his role in helping her to get better. He involved himself in her treatment by asking her about DBT and practising the skills with her. He attributed her reduced symptoms to “her DBT, her training, and the fact that I am her integral, stable foundation.” He believed that she needed: “…someone who won’t get triggered by her getting triggered. So, when she has an episode … it was very unhelpful for her if I would also get upset … so that’s why I mean stable - not reactive.” Dylan (D1; w/o BPD), Paul (D3; w/o BPD) and Sean’s (D2; w/o BPD) understanding of responding to their partners’ distress without ‘reacting’ likely promoted positive communication behaviours and an increased feeling of closeness and security in their partners with BPD.

Another symptom of BPD in these relationships was fear of abandonment. Three partners with BPD - Josie (D1), Lisa (D4), and Sarah (D5) - noticed that early on, they struggled to give their partner the freedom to have their own space; however, they reported having worked towards this. Lisa explained: “There was a time when I could not be separated from him … I’ve learned to have my own time, and space, and occupations that are meaningful to me and grow my own identity.” Lisa’s use of the phrase “grow my own identity” reflects increased confidence, self-esteem and a developing sense of self. Josie expressed a similar progression:

“…I was like, if I give him space now he might leave later … so I was very much in the state of mind that I need to keep him with me at all times because eventually he will be gone, because I was so used to that. But now I’m like ‘oh, weird, he’s not going anywhere!’ (laughs).”

Once the female participants were able to recognise that their dislike of separation was a symptom of their fear of abandonment, the recognition that their partners were indeed committed (“oh, weird, he’s not going anywhere”) helped them build some tolerance to brief separations.

Each couple spoke about how they responded when fear of abandonment arose in their relationship. Brent (D6; w/o BPD) related that Casey (D6; BPD) sometimes had “the gut feeling that everything will fall apart”. Donna (D3; BPD) explained, “when I’m really angry and I want to leave him, I’m actually afraid that he’s [Paul, D3; w/o BPD] going to leave me.” Lisa (D4; BPD) explained that she kept – “…asking him [James, D4; w/o BPD], “do you still love me?” Tina (D2; BPD) admitted she was “…quietly vigilant about not allowing anything to cause that to happen.” While they all grappled with this fear, four men reported having learned ways to calm their partner. Brent assisted Casey by – “I just have to say, ‘look at the moment, there would be no reason for that to happen,’ and I just give examples, and say think about the future, be reassuring.” For Josie (D1; BPD), while the fear surfaces less often now, it is not completely gone:

“… he forgets that that is so programmed for me.…it used to be … like a daily thing and he’d have to reassure me … And I think he thought it reflected on him that I thought he was a flaky person, but he just got used to the fact that no, that’s just the BPD speaking for me basically. So, it’s got better over time.”

Fear of abandonment is underpinned by anxious attachment. Between Sarah (D5; BPD) and David (D5; w/o BPD), Sarah scored higher on anxious attachment on ASQ-SF. She shared that David used to take her fears as a personal slight against him but now feels - “He’s got much better at that now, now that he doesn’t take it as a personal ‘you’re not responding to what I need.’” Tina (D2; BPD) feared abandonment frequently in the past and explained that Sean (D2; w/o BPD) would give her verbal reassurance that he was “not going anywhere … He had a lot of people asking him, why do you stay, and … he couldn’t understand why … because his wife is just unwell.” While the male partners struggled to understand their partners’ fear of abandonment, partners with BPD learned to engage in open communication about their fear. Despite frustrations, male partners were able to regulate themselves and their partners with reassurance and by “prov[ing] [them] wrong.” The secure base provided by partners without BPD likely influenced the openness exhibited by partners with BPD. Consequently, the participation of partners without BPD in these interactions led to mutual recognition of signals and the development of a secure and integrated relationship.

## 4 Discussion

The current study extends current literature by examining attachment styles in the context of romantic relationships of people with BPD, and the role of the partner without BPD in creating a secure attachment. This research also augments what is known about conflict and caregiving in romantic relationships involving people with BPD, revealing a profile of destructive conflict behaviours (e.g. reactivity) and suboptimal caregiving behaviours (e.g. mis-attunement to partner’s distress) that can be effectively targeted through treatment. The study offers unique findings, describing drivers of satisfaction and longevity in romantic relationships involving couples where one partner has BPD. These findings offer a hopeful message to people with BPD and identify collaborative treatment targets for them, their partners, and clinicians who work with them.

### 4.1 Through an attachment lens

Participating couples acknowledged challenges navigating the symptoms and impact of BPD within their relationships; however, they also shared common goals and capacities that underpinned the success of their relationships. They highlighted the importance of communication, psychological strategies, and skills for regulating emotions and co-regulating distress. Specifically, all participants reported applying DBT skills to self-soothe, communicate clearly, and find pragmatic solutions during their relationship challenges.

According to Bowlby’s attachment theory, while the attachment system is pivotal in early life, it also impacts one’s behaviour within adult relationships (46). Early experiences with caregivers give way to the development of an internal working model of attachment in children, which influences their expectations, trust and behaviour in future social and romantic relationships throughout life. Attachment figures are people we seek proximity to when we are stressed or unwell, needing comfort and support, or when we are seeking a secure base from which to understand ourselves and others (46–48). An attachment figure who is ‘psychologically available’ most of the time can generate the formation of a secure attachment. Within the context of this study, participants with BPD saw their partners as a secure base when they felt distressed, with the w/o BPD participants perceiving themselves as part of that social transaction and capable of affecting change in their partners’ responses through their own behaviour. These perceptions suggest a psychological availability that aligns with Bowlby’s theory of attachment (7, 46) and explains the improvements reported by participants with BPD over the course of their relationships.

### 4.2 Evidence of secure base behaviours

Adults who are securely attached are more capable of seeking and providing support than are insecurely attached adults. ‘Secure base behaviours’ are known to assist in restoring the distressed adult to emotional stability (18, 49). Interestingly, despite ASQ-SF scores for all participants falling within the insecure attachment range (refer to Supplementary Materials), couples used secure base behaviours with one another. This was evidenced by mutually open communication and the willingness of participants with BPD to share difficult feelings with their partner to elicit comfort and resolution.

It is notable that at times, participants’ descriptions of themselves and their partners reflected probable power dynamics within their relationships. This was evident from narratives of several partners without BPD and could be interpreted as their adopting a caring or supportive role as a well-intentioned response to their partner’s BPD-related difficulties. However, partners with BPD also described having agency and initiative when their partners without BPD were struggling; they supported their male partners through adversity and expected reciprocal effort and commitment.

Across the sample, couples described learning and practising skills that mutually supported the relationship, alleviated distress, and helped resolve conflict. Despite their varied abilities and approaches, all w/o BPD participants demonstrated proficiency in offering support to their partners, including allaying their partners’ fears of abandonment. Male participants who had an anxious attachment style were more effective at giving and receiving emotional comfort and engaging in interactions with their partners than were men with an avoidant attachment style. While the former demonstrated the desire and ability to engage with their partner when she communicated distress, men with an avoidant attachment style opted to provide space rather than closeness in such situations. This contrasts with literature suggesting that adults with an anxious attachment style do not differ from those with an avoidant attachment style in their ability to give and receive comfort (49). Notably, no such difference was observed between anxious and avoidant women in this study.

### 4.3 Social transactional model of relationships

The social transactional model emphasises how individuals and their social environment continuously influence one another over time, with each response becoming part of the context for the next (50, 51). In line with the concept of ‘partner effects’, the transactional model can be explained by each partner’s behaviour influencing the other partner’s responses, which shapes subsequent behaviour and the overall interaction pattern. For example, one partner may express distress or a need for reassurance; if the other responds dismissively, the distressed partner’s arousal may escalate, and they may intensify their behaviour to be noticed. If escalation is then met with increased attention, soothing, or capitulation, the escalation is inadvertently reinforced as an effective strategy, and the other partner may also be shaped toward “placating” to end the conflict. Over time, these repeated transactions can stabilise into a characteristic relationship style that maintains dysregulation and conflict.

Applied to BPD, this framework is consistent with the idea that emotion dysregulation develops and is maintained through transactions between emotion vulnerability and invalidating responses from close others (50, 51). Clinically, the implication is that improving outcomes often requires shifting both sides of the interaction: supporting the person with BPD to accurately label and express internal experiences and to use regulation skills, while supporting the partner to respond with attunement and validation and to avoid reinforcing escalation.

Insights from this study suggest that a romantic partner can actively contribute to the success of the relationship when one partner has BPD. Two of the men in this study learned and practised DBT skills, assisting their partners to utilise specific skills when distressed. Men not as actively involved in DBT developed their own distress mitigation strategies in discussion with their partners.

The study findings validate the social transactional model and highlight the importance of a healthy transactional approach in relationships where one person has BPD. It recognises that partners without BPD require more than psychoeducation, including but not limited to up-skilling in communication, emotion regulation, and validation (50). Secure base behaviours of the partner without BPD enable the symptomatic person to test new or learned skills and receive feedback within their relationship. Additionally, the person with BPD can respond to interpersonal triggers by practising emotional regulation, self-soothing, and interpersonal skills, while their partner practices attunement, validation, and problem-solving. In this way, the likelihood of consistent, healthier transactions increases as they continually shape their attunement to each other’s needs (51).

### 4.4 Earned security in BPD

Open communication is often a feature of securely attached relationships (12). Despite all participants (with and without BPD) individually exhibiting an insecure attachment style, open communication was highlighted by the couples as a contributor to the formation of a ‘secure attachment’ and their relationship longevity. This can be attributed to the development of ‘earned security.’

The phenomenon of shifting one’s attachment style from insecure to secure in later life is known as ‘earned security’ (52). A requirement for this is having the ability to mentalise within the context of attachment relationships, and reflect on our own and others’ thoughts, feelings, and behaviours, otherwise known as ‘reflective functioning’ (53). Consistent with the social transactional model, accurate expression of emotions or thoughts by the w/o BPD partner improved the symptomatic person’s ability to reflect on their own as well as their partner’s thoughts, feelings, and behaviours (51). Consequently, earned security could be attained through: 1) Meta conditions (e.g. “being intentional” and “having surrogate attachment figures” including friends, therapists, and spouses), 2) Making intrapsychic changes (e.g. “redefining identity and worth” and “relinquishing the victim mentality”), and 3) Making interpersonal changes (e.g. “making peace with the past” and “taking small risks with trust”) (54). Participants diagnosed with BPD all demonstrated progress towards achieving elements of this model, with their partners fulfilling the role of surrogate attachment figures and helping them to make intrapsychic and interpersonal changes.

Josie, whose attachment score was closest to being secure, may best illustrate this. At the time of the interview, she was the least symptomatic participant and considered herself to be in remission since starting her relationship with Dylan 18 months previously. She credited her remission to completing DBT and the support of her partner. She described trusting Dylan to be there for her, and that she could go to him with “anything”. She also felt that having a “solid” partner gave her someone to practice her skills with and that she effectively “needed” that relationship to get well. Her case is an example of how a combination of relational skills (communication, interpersonal, and emotion regulation) and the transactional opportunity to practice those skills in real-life situations (with a partner who is a “solid” surrogate attachment figure) can help shift an insecurely attached person towards security.

### 4.5 Strengths and limitations

The self-report ASQ-SF (17) was chosen for its reported reliability and validity for measuring overall attachment style. It was interpreted as reflecting early experiences and attachment orientation before entering the relationship, but not necessarily participants’ attachment to their current romantic partner. Consequently, our study’s findings indicate that people with insecure attachment histories in childhood may be able to ‘earn security’ and develop satisfying secure attachments in adulthood.

The study focused on couple dyads, with each partner providing an interrelated account situated within a shared relational context. This added analytic complexity and supported greater contextual interpretative depth and rigour. Accordingly, although participants described their experiences individually, each couple’s accounts were interpreted cumulatively.

IPA’s strength lies in its transferability of findings to similar relational contexts (e.g., heterosexual couples where female partners have BPD and both partners may have insecure attachment styles). Since IPA uses a purposive and idiographic sampling approach and prioritises rich interpretations of accounts from a relatively small, homogenous sample rather than seeking to make claims about a broader population, future research would benefit from extending the study’s findings. The findings also reflect a DBT-based skills approach to relationship health, which is likely a consequence of previous treatment with DBT (the most commonly available manualised treatment nationally). Other BPD-appropriate psychotherapies also focus on relationships and interpersonal difficulties; therefore, the findings of this study need to be explored more broadly in the context of other treatment modalities. Future research may also consider evaluating group therapy modules for couples while concurrently examining changes in secure base behaviours over time.

### 4.6 Clinical implications

Findings from this study have practical implications for clinicians working with people with BPD who are in romantic relationships or hoping to be. Firstly, they validate the therapeutic value of modelling secure base behaviours with clients and demonstrating relational skills implicitly and explicitly. Recognising and honouring a client’s effort to change, practising skills with them, and retaining faith in a person’s agency and competency to improve, can demonstrate ‘secure base’ behaviours that encourage autonomy and growth. Being calm and consistent in the face of a client’s powerful affect and providing a safe, reliable space where the client feels validated can help them attain earned security.

Combining skills-based training, psychoeducation, carer/family support, and coaching with attachment-specific behaviours such as attunement, validation, co-regulation, and calming fears of abandonment may assist people who have partners with BPD to recognise and respond to their partner’s needs. This systematic approach can promote more satisfying outcomes in the romantic relationships of people with BPD, which are widely believed to be a foundation for recovery and ‘having a life worth living’ (35, 55, 56).

## Data Availability

The original contributions presented in the study are included in the article/supplementary material. Further inquiries can be directed to the corresponding author.
